# Origin of Medical Journals

**Published:** 1840-01

**Authors:** 


### *Origin of Medical Journals.*

In the autumn of 1796, Dr. Elihu H. Smith, a man of much refinement and rare acquirements in the profession, conceived the project of a medical periodical. He communicated his plan to his friend Dr. Edward Miller, and they together laid the design before Dr. Samuel L. Mitchell, who had just returned from Europe. They all three united in the enterprise and pursued it with so much vigour, that in August, 1797, the first number of the New York Medical Repository made its appearance. This work, the first of its kind, was warmly received, and ably supported. In its pages are to be found valuable records of our epidemics, and the other diseases of this country. Beginning with the yellow fever of 1793, it contains the complete history of that disease up almost to its last appearance here. It contains the most ample records of the spotted fever, a very fatal disorder that prevailed in the northern and eastern parts of the United States, from about 1807 until after the late war. It contains valuable memoirs

*We must not omit to mention that the putrefaction of the body was already considerably advanced, when the examination took place.

on the typhoid pneumonia, that committed such havoc among our soldiers, and spread to different parts of the country during the years 1813 and '14, and for a short time afterwards. It is rich in medical topography, and in the chemistry of the day, on which latter subject the celebrated Priestly was one of its diligent contributors.

From this as a parent stock, says the Rev. Dr. Miller, have sprung a number of similiar works in Europe and America. "The Medical and Philosophical Journal of London was commenced soon after the appearance of the Medical Repository, with the avowal of its editor that he took the hint from New York. Other editors in London, Paris, Edingburgh, and Bremen, in a short time started similar journals.—*New York Journal of Medicine and Surgery.*

